# Open, single-blind, double-blind: which peer review process do you prefer?

**DOI:** 10.1186/2050-6511-15-55

**Published:** 2014-09-30

**Authors:** Elizabeth C Moylan, Simon Harold, Ciaran O’Neill, Maria K Kowalczuk

**Affiliations:** 1 BioMed Central Ltd, 236 Gray’s Inn Road, London WC1X 8HL, UK; 2 Present Address: Nature Communications, The Macmillan Campus, 4 Crinan Street, London N1 9XW, UK

## Abstract

*BMC Pharmacology and Toxicology* was created from the merger of two journals within the *BMC* series published by BioMed Central: *BMC Pharmacology* and *BMC Clinical Pharmacology. BMC Pharmacology* operated anonymous peer review whereas *BMC Clinical Pharmacology* operated a fully open peer review policy where the identity of the reviewers was known to the editors, authors and readers. The merged journal also adopted a fully open peer review policy. Two years on we discuss the views and experiences of our Editorial Board Members towards open peer review on this biomedical journal.

## The story so far

The *BMC* series of journals was established in 2000 by BioMed Central to provide open access to research published across a range of disciplines in biology and medicine [1]. Over the years, new journals have been launched to fulfil a particular research need (*BMC Veterinary Research* 2005, *BMC Systems Biology* 2007 and *BMC Psychology* 2013 are among such examples). All the biology journals within the *BMC* series operate traditional anonymous peer review, where the authors do not know who the reviewers are. However, the medical journals were established with open peer review, where the identity of the reviewers is known to all parties – editors, authors and readers [2].

Two years ago the pharmacology titles in the *BMC* series, *BMC Pharmacology* (a biology journal) and *BMC Clinical Pharmacology* (a medical journal)*,* joined forces under the new title *BMC Pharmacology and Toxicology*[3]. The combined journal retained the full scopes of the original titles while also expanding to explicitly include the field of toxicology. Many of the original Editorial Board Members remained associated with the journal, and new academics and clinicians also joined.

In merging a biology journal operating anonymous peer review with a medical journal operating open peer review, we debated what the peer review process should be on *BMC Pharmacology and Toxicology*. In the end, the journal naturally retained the open peer review policy previously adopted by *BMC Clinical Pharmacology* – in keeping with all the other medical titles in the *BMC* series. *BMC Cancer*, another journal in the *BMC* series with biology and medicine disciplines, also operates open peer review.

Under open peer review, authors know who reviewed their manuscript (reviewer reports are signed) and, if the manuscript is published, the reader will also see the reviewers’ comments and the authors’ response. These comments are published as part of the ‘pre-publication history’ accompanying the published article, which also contains all versions of the manuscript and (where relevant) editors’ comments. By making the peer review process completely transparent we aim to reduce the competing interests that can occur especially for a journal which frequently publishes research sponsored by pharmaceutical companies [4]. See this recent article [5] for an example of a pre-publication history [6].

While many have recently discussed the benefits of open peer review including transparency, accountability and giving credit to reviewers [7-11], there are challenges too. Potential reviewers may be more likely to decline to review [12] and some (junior) reviewers may be reluctant to sign a critical report [13,14]. There are field-specific differences too. Medical disciplines with the particular need to be transparent about treatments for patients and competing interests appear to be more willing to embrace open peer review than the biological sciences. However, within biology there are differences between research fields, too: for example, the bioinformatics and genomics communities seem to accept open peer review more readily than traditional subjects such as immunology and physiology [15]. Perhaps this reflects their familiarity with features of open-source software and social-networking technologies [16].

At the time of the merger of *BMC Pharmacology* and *BMC Clinical Pharmacology,* we said that we would report on our findings of open peer review with the resulting biomedical journal. So, two years down the line, how have things fared with *BMC Pharmacology and Toxicology*?

## Our survey says…

The Editorial Board Members on *BMC Pharmacology* & *Toxicology* represent a broad demographic from which to solicit views on open peer review given their variety of expertise in biology and clinical disciplines. Therefore, we surveyed the current members of the Editorial Board with eight questions (see Table 1). These covered their particular expertise and whether they preferred one system of peer review over another as an author, reviewer, or handling editor. Table 2 gives a summary of the main systems of peer review that were included for the purpose of this survey. We also asked whether our Editors read the pre-publication histories which accompany published articles. Finally, any other comments on open peer review were welcomed.

**Table 1:** **Survey questions to the *BMC Pharmacology & Toxicology *** **Editorial Board**

**Number**	**Questions**
1.	Is your area of expertise in medicine or biology?
	If medicine, are you a clinical academic or full time academic?
2.	How many years have you been working as an academic?
3.	Choose one that best describes you:
	I was on the original board of *BMC Clinical Pharmacology*
	I was on the original board of *BMC Pharmacology*
	I joined the editorial board of *BMC Pharmacology and Toxicology* within the last 2 years.
4.	As an **author**, have you published in an open peer review journal? (meaning that the reviewers’ identity was revealed to you as an author)
	4a (if yes) Do you think reports were less/equally/more useful to you than those from a closed peer review journal? If you have never published in a closed peer review journal please go to the next question.
	4b (if no) Would you consider publishing in an open peer review journal? (if no, why?)
5.	As a **reviewer** which peer review system do you prefer, and why?
	5a Open (authors and reading public know reviewers’ identity)
	5b single-blind (i.e. reviewers know authors’ identity but not vice versa)
	5c double-blind (i.e. authors and reviewers do not know each other’s identity)
6.	As a handling **editor** do you prefer a different (from your answer to question 5) model of peer review?
	6a (If yes), which model do you prefer and why do you have a different preference as an editor compared to as a reviewer?
7.	As a **reader** do you look at the pre-publication histories on *BMC Pharmacology and Toxicology* (or any of the open peer review journals in the *BMC* series)?
	7a If no, why not?
	7b If yes, what is your main reason for looking at the pre-publication history?
8.	Do you have any further comments on open peer review?

**Table 2:** Definitions of open, single-blind and double-blind peer review as operated by BioMed Central

Open peer review	Editors, authors and reviewers know each other’s identity. If the manuscript is published, the reviewer reports, any editors’ comments, authors’ response and all versions of the manuscript are available via an accompanying ‘pre-publication history’.
Single-blind peer review	Reviewers know authors’ identity but not vice versa.
Double-blind peer review	Authors and reviewers do not know each other’s identity

**Figure 1:**
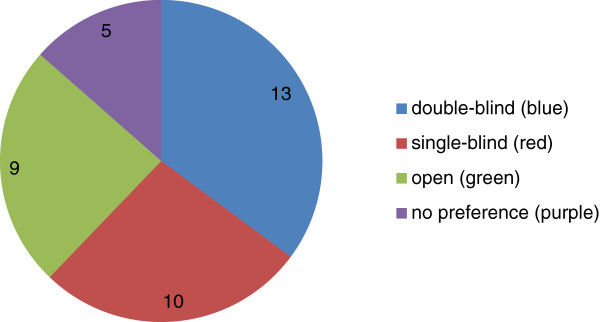
Piechart of the responses received from the Editorial Board to the question: ‘As a reviewer which peer review system do you prefer?’

Of the 83 Editorial Board Members approached we received 37 replies (a response rate of 45%). Half the replies were from medics (of whom a third were practicing clinicians) and half were from non-medical fields including biology, chemistry and pharmaceutical sciences. The anonymised answers are provided in Additional file 1.It is clear that the Editorial Board Members who did respond were consistent in that, regardless of their potential role as ‘author’, ‘reviewer’ or ‘editor’, they prefer the same system of peer review. It was not the case that Editorial Board Members preferred one type of peer review system in their role as editor, and another in their role as reviewer (for example). Somewhat surprisingly, the majority of Editorial Board Members preferred double-blind peer review over open peer review (see Figure 1). Among the reasons put forward in support of double-blind peer review was the fact that this was perceived to be the most objective system and thus minimized bias. However, Editorial Board Members appreciated that it may not be very effective in practice as it may be possible to infer from the methods used, or reference list, who the authors are.

**Figure 2:**
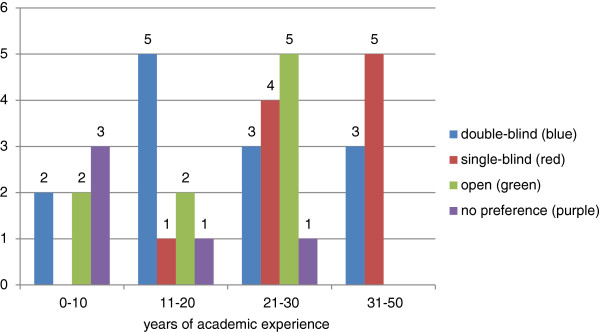
Diagram of the preferences for a given peer review model classified by years of academic experience.

Although some Editorial Board Members recognised the value of open peer review, commenting that it is more egalitarian, increasing accountability and transparency, it was felt that open peer reviews may be more difficult for early career researchers to provide - as has been noted again recently [17]. Our survey suggested that the more early career researchers preferred double-blind peer review while the more senior Editorial Board Members preferred single-blind or double-blind models (Figure 2).

**Figure 3:**
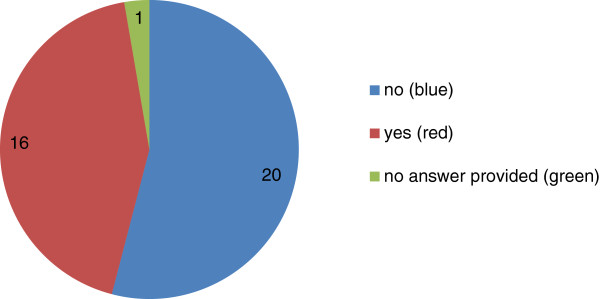
Piechart of the responses received from the Editorial Board to the question: ‘As an author, have you published in an open peer review journal?’

In their role as authors, approximately half of the Editorial Board had published in an open peer review journal (see Figure 3). The majority found that the reports they received during an open peer review process were *equally* useful to those received under anonymous peer review. However, in previous research [18] we have found that the quality of reports received was slightly higher under open peer review than anonymous peer review. Reviewers under an open peer review system provided more feedback on the methods, more constructive comments on the content and substantiated their feedback better with explicit evidence [18].

**Figure 4:**
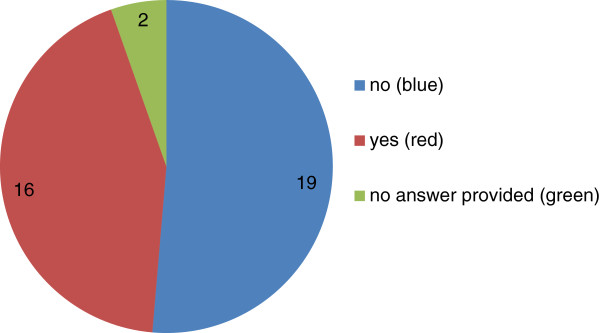
**Piechart of the responses received from the Editorial Board to the question: ‘As a reader do you look at the pre-publication histories on *BMC Pharmacology and Toxicology *** **(or any of the open peer review journals in the *BMC *** **series)?’**

Finally, approximately half of the respondents of this survey read the pre-publication history which accompanies open peer reviewed articles in the *BMC* series, including *BMC Pharmacology and Toxicology* (see Figure 4). The pre-publication history contains all versions of the manuscript, named reviewer reports, author responses and (where relevant) editors’ comments. Of those Editorial Board Members who reported that they did not look at the pre-publication history, many simply had no time or inclination to do so or wanted to judge an article on its own merits. Some simply did not appreciate this information was provided. However, of those that did look, many did so in order to determine the scientific and ethical credentials of the reviewers and for further insights into peer review.

The survey has provided a sample of the views and experiences of academics and clinicians with respect to peer review on a biomedical journal, and as a result we appreciate there are improvements which can be made. However, this small survey has some limitations too. The low response rate limits the generalizability of the findings; we were not able to draw any specific conclusions reflecting (for example) the attitudes of particular groups based on gender/subject background/seniority towards different systems of peer review.

## What next?

So two years into the open peer review experiment on *BMC Pharmacology and Toxicology,* will we continue to operate an open peer review system? Put simply: yes.

Among the Editorial Board Members who responded to our survey, there appears to be an overall preference for a peer review system that is double-blind (where authors and reviewers are not revealed to each other). Other much larger surveys have also come to similar conclusions [17,19,20]. However, it is unclear if this is a genuine feeling among researchers or rather ‘wishful thinking’ that double-blind peer review intuitively seems the fairest approach [21]. Certainly not many biology and medical journals operate double-blind peer review and from a pragmatic view point it is difficult to prevent reviewers from guessing who the authors are.

We will continue with open peer review at *BMC Pharmacology and Toxicology* because of the ethical grounds for doing so [2] and because the potential benefits outweigh the negatives [22,23]. Open peer review provides a fully transparent pre-publication history, and the reading public can see *who* reviewed the manuscript and *what* was said. Having access to peer reviews also provides valuable information for training purposes [24,25] and allows further research into the benefits of peer review [26]. And by making peer review completely open and reviewers (and editors) accountable, we aim to reduce the competing interests that can occur. But perhaps more relevant, in this era of ‘predatory publishers’ [27] and ‘sting’ operations [28] open peer review ensures that the decision-making process is fully transparent for all to see.

From the feedback we receive more generally from authors on our open peer review journals in the *BMC* series, many value the helpfulness, quality and detail of the reports. But given the responses received from our Editorial Board Members we need to make more of the fact that the pre-publication history is provided for readers and what it contains.

If you have any further feedback on the open peer review policy operated by *BMC Pharmacology and Toxicology* we’d certainly welcome your comments.

## Competing interests

ECM, CON and MK are employed by BioMed Central. SH is employed by Nature Publishing Group.

## Authors’ contributions

SH and CON designed the survey. EM and MK collated the data. MK analysed the data. EM wrote the first draft and revised the text. All authors contributed to the writing of the manuscript and approved the final version.

## Authors’ information

ECM is the Biology Editor at BioMed Central. SH is an Associate Editor at *Nature Communications*. CON is Associate Publisher at BioMed Central and Editor of *Biome*. MK is the Deputy Biology Editor at BioMed Central.

## Pre-publication history

The pre-publication history for this paper can be accessed here:

http://www.biomedcentral.com/2050-6511/15/55/prepub

## Supplementary Material

Additional file 1 Anonymised survey responses from the Editorial Board. Click here for file
